# Late postnatal steroid treatment using oral betamethasone can help to close ductus arteriosus in extremely preterm infants who cannot be weaned from ventilation

**DOI:** 10.1007/s00431-024-05840-9

**Published:** 2024-11-28

**Authors:** Aude Remy, Marine Vincent, Blandine Pastor-Diez, Jean-Charles Picaud

**Affiliations:** 1 Neonatology, University Hospital Croix Rousse, 103 Grande Rue de La Croix Rousse, 69004 Hospices Civils de LyonLyon, France; 2 Service de Reanimation Néonatale du Centre Hospitalier Universitaire de Tivoli, La Louvière, Belgium; 3 CarMen Laboratory, INSERM, INRA, Claude Bernard University, Lyon 1, 69310 Pierre-Bénite, France

**Keywords:** Bronchopulmonary dysplasia, Prematurity, Patent ductus arteriosus, Corticosteroids, Ventilation

## Abstract

Late postnatal steroids are given to premature infants who cannot be weaned from ventilation because of the possible development of bronchopulmonary dysplasia (BPD). At that time, some infants still have a patent ductus arteriosus (PDA). In our experience, the use of betamethasone (BTM) seems to reduce the need for surgical/endovascular treatment of PDA. We evaluated herein the impact of oral BTM on PDA in extremely preterm infants with BPD. Extremely preterm infants (GA < 29 weeks) with PDA and treated with BTM to facilitate extubation/avoid reintubation were included in this retrospective, single-centre study. BTM was administered orally at 0.3 mg/kg/day for 3 days, 0.15 mg/kg/day the following 2 days, and 0.05 mg/kg/day on the last day. An echocardiography was performed before and after BTM treatment. The 51 infants included were born at a median [IQR] GA of 25.7 [25.0–26.7] weeks. At the time of BTM treatment (28 [26–30] days), 94.1% (48/51) were on invasive ventilation, and most (44/48, 91.7%) were extubated after BTM treatment. At that time, nearly all infants had a closed or non-haemodynamically significant PDA (50/51, 98.0%). None required surgical or endovascular treatment after BTM. Adverse effects included transient moderate hypertension (68.6%), transient hyperglycaemia (15.7%), and transient slowing of postnatal weight gain during BTM treatment.

*Conclusion*: In extremely preterm infants with a severe respiratory condition at 3 weeks of life, oral BTM treatment can help wean invasive ventilation and is associated with PDA closure. It could reduce the need for surgical or endovascular treatment that are associated with serious adverse effects.

*Trial registration:* Clinicaltrials.gov NCT05987202.

**Table:** 

**What is Known:**
*• Patent ductus arteriosus and bronchopulmonary dysplasia are two most frequent complications of extreme prematurity.*
*• Betamethasone is one of the corticosteroids used to help wean invasive ventilation in infants at risk for bronchopulmonary dysplasia.*
**What is New:**
*• In extremely preterm infants still ventilated after 3 weeks of life and suffering from patent ductus arteriosus, treatment with oral betamethasone facilitated ventilatory weaning*
*• Oral betamethasone treatment was associated with patent ductus arteriosus closure in almost all infants.*

## Introduction

Patent ductus arteriosus (PDA) affects 20 to 50% of infants born before a gestational age (GA) of 32 weeks and up to 60% before 29 weeks [1]. It is associated with increased mortality and morbidity: prolonged ventilatory support, pulmonary haemorrhage, necrotising enterocolitis (NEC), and intraventricular haemorrhage (IVH) [2]. Medical treatment (non-steroidal inflammatory drugs, paracetamol) results in PDA closure in 67.4% of preterm infants [3]. When medical treatment fails or is contraindicated, surgical or endovascular treatment may be considered. All of these treatments are associated with a risk of severe complications [3].

Bronchopulmonary dysplasia (BPD) affects 26.5% of preterm infants born between 24 and 29 weeks in Europe and 42.6% have severe BPD [4]. It is diagnosed from mild to severe according to ventilatory support and oxygen dependency at 28 days of life and 36 weeks corrected GA [5], but it can begin around 2 to 3 weeks of life and manifest as difficulty weaning the child from invasive ventilation [2, 6, 7]. Treatment with systemic steroids can be used when ventilatory weaning is impossible after the third week of life [6–8]. Steroids are used to facilitate extubation [6] or avoid reintubation due to the severity of BPD [9]. The main product used is dexamethasone (DXM), which reduces the risks of mortality and BPD, but has been associated with increased risk of impaired neurodevelopment.

Steroids could possibly have a direct effect on the PDA leading to closure through interference with prostaglandin synthesis [10] or a reduction sensitivity of the ductal muscle to prostaglandin E2 [11]. It has also been reported that postnatal DXM was associated with PDA closure [12–16]. However, due to long-term side effects of DXM, other products are used for the treatment of BPD, such as hydrocortisone and betamethasone (BTM). Hydrocortisone had no effect on the course of PDA when administered late (after 7 days of life) [17], but was associated with reduced incidence and need for surgical treatment of PDA [18, 19] when administered early. Concerning BTM, it has been reported in foetal rats that BTM induces ductal constriction [20]. Furthermore, infants born from mothers who received BTM antenatally had less frequently PDA than those born from untreated mothers [21, 22], and postnatally, BTM has a vasoconstrictor effect on the PDA [23] and could contribute to PDA closure. In our experience, the use of BTM seems to reduce the need for surgical/endovascular treatment of PDA. We therefore aimed to evaluate the impact of oral BTM on PDA closure in a population of extremely preterm infants at high risk for BPD.

## Methods

### Study design

Retrospective, single-centre study.

### Population

All infants born < 29 weeks GA and hospitalised between 1 January 2018 and 31 December 2022 in the tertiary care unit of the Croix Rousse University Hospital (Lyon, France), were eligible if they met the following inclusion criteria: patent PDA and postnatal steroids treatment for invasive ventilation and/or non-invasive ventilation with prolonged oxygen dependence and/or hypercapnia.

### Postnatal steroids

BTM treatment was given to infants with severe respiratory condition at 3 weeks of life (i.e. remaining on invasive ventilation) with the aim to wean them off ventilation, as well as those presenting high capnia (> 9 kPa) and/or FiO2 (> 0.4) under non-invasive ventilatory support with the aim to avoid reintubation. The use of BTM was based on previously published data from our unit, and consisted of 6 days of oral administration: 0.3 mg/kg/day for 3 days, 0.15 mg/kg/day the following 2 days, and 0.05 mg/kg/day on the last day [24]. In accordance with national recommendations, administration was initiated around the 21st day of life [6]. The treatment was indicated in infants with failure to wean invasive ventilation (failure of extubation or deventilation), infants with refractory hypoxia, and infants with non-invasive ventilation (NIV) to avoid re-intubation when there were significant hypercapnia (> 9 kPa) and hypoxia (FiO_2_ > 0.4).

### Treatment of PDA

The first-line treatment of PDA was Ibuprofen. Paracetamol was used when there was a relative contraindication to ibuprofen (clinical bleeding, thrombocytopenia < 50,000 platelets/mm3, liver failure, diuresis < 1 ml/kg/hour over the last 12 h, uncontrolled sepsis, digestive intolerance).

### Collected data

Data concerning pregnancy, delivery, infants at birth, and postnatal evolution were collected. BPD was considered as present when ventilatory support or oxygen therapy was needed at 36 weeks of postconceptional age (PCA) and severity was classified according to Jobe et al. [5]. Postnatal steroid treatment with BTM was also collected: duration of the course, number of courses, total cumulative dose.

To evaluate the effect of BTM treatment on respiratory support we collected information about respiratory status at the beginning and 24 h after the end of BTM treatment. For those on invasive ventilatory support BTM treatment was considered effective when they were extubated; for those on non-invasive ventilatory support, we collected capnia and FiO2 at the beginning and 24 h after the end of BTM treatment, and BTM was considered effective when FiO2 was reduced to < 0.3 and capnia was reduced < 8 kPa.

We evaluated adverse effects of BTM: systemic hypertension, hyperglycaemia, poor growth, late-onset sepsis (LOS), and NEC. LOS was considered present when infection was proven [25]. NEC was classified according to the modified Bell’s classification. We collected the interval between the end of BTM treatment and the occurrence of LOS as well as NEC. Systemic hypertension was considered present when mean blood pressure was > the 95th percentile for ≥ 48 h and treated if high blood pressure persisted for more than 96 h [26]. Hyperglycaemia was considered to be present when capillary blood glucose was ≥ 10 mmol/L or ≥ 8 mmol/L in the presence of glycosuria. In such cases, insulin was initiated. In infants already on insulin, this was increased if capillary blood glucose was ≥ 9 mmol/L. Weight gain was calculated the week before, during and the week after BTM treatment using the following equation: W2-W1/ ([W1 + W2]/2)/n), with n corresponding to the number of days between the measurement of body weight at day 1 (W1) and at the end (W2) of the treatment. Z-scores for body weight were calculated with reference to Fenton’s curves at the first and last day of BTM [27]. Delta z-scores for body weight between the beginning and 36 weeks PGA were calculated.

To evaluate the effect of BTM treatment on PDA, we collected data from echocardiography performed before and after BTM treatment, and PDA was classified according to McNamara et al. [28]. It was considered haemodynamically significant when the pulsatile transductal flow was maximum velocity < 2 m/s or continuous and at least one of the following criteria were present: ductal diameter > 1.5 mm, left atrium to aorta ratio > 1.5 (presence of signs of left volume overload), zero or negative diastolic perfusion in the systemic arteries (negative or zero diastole, resistivity index > 0.8) [28]. It was considered that BTM had a positive impact on the PDA when it was closed or hemodynamically insignificant after BTM treatment.

### Statistical analysis

Results were expressed as numbers (percentage) for discrete variables and medians [interquartile range, IQR] for continuous variables. The Chi-squared test was used to compare qualitative variables, and the Wilcoxon test to compare quantitative variables. Statistical analyses were performed using the R Studio program version 4.2.1 (Inc., Boston, MA, US). *P* < 0.05 was considered statistically significant.

### Ethics

The study was conducted in accordance with the Declaration of Helsinki. The study was approved by the Institutional review board (*Comité scientifique et éthique des Hospices Civils de Lyon*, IRB: 00013204: n°23_178), the national data protection commission (CNIL) (n°23_5178) and registered on ClinicalTrials.gov (NCT05987202).

## Results

**Fig. 1:**
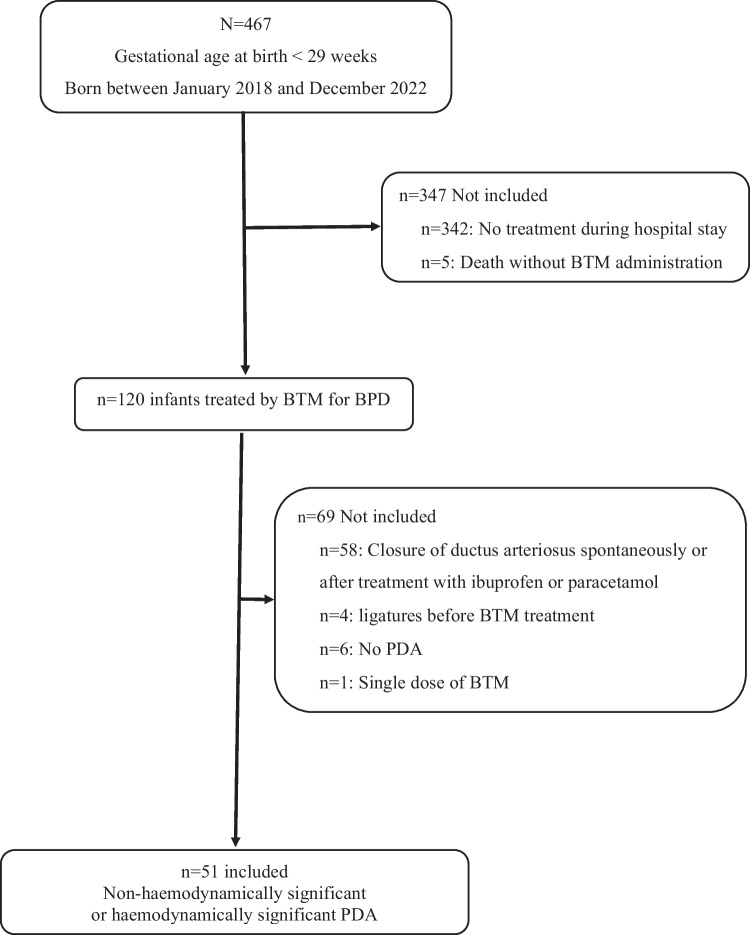
Flow chart. BTM, betamethasone; PDA, patent ductus arteriosus

Among the 467 extremely preterm infants admitted between January 2018 and December 2022, 25.7% (120/467) received BTM. Of these, 42.5% (51/120) presented with PDA and were included (Fig. 1); the PDA was hemodynamically significant in most infants (80.4%, 41/51).

At birth, the median [IQR] GA was 25.7 [25.0–26.7] weeks and birth weight was 720 [640–798] g. Before BTM treatment administration, most infants received ibuprofen and/or paracetamol (88.2%, *n* = 45; of whom 14 [31.1%] received both ibuprofen and paracetamol). 17/51 received a single course of ibuprofen, 28/51 received two courses of ibuprofen, and 14/51 received a course of paracetamol, given the relative contraindications to ibuprofen.

Postnatal growth failure occurred in 22.0% (*n* = 11) of infants (Table 1).

**Table 1:** Clinical characteristics of the 51 premature infants with bronchopulmonary dysplasia and patent ductus arteriosus treated with oral betamethasone

	Total population, *N* = 51
At birth	
Antenatal steroids (full course)	38 (74.5)
Caesarean section	33 (64.7)
Gestational age, weeks	25.7 [25.0–26.7]
Birth weight, g	720 [640–798]
Birth weight < 10th percentile	9 (17.6)
Male sex	25 (49.0)
Between birth and postnatal betamethasone treatment	
Invasive ventilation, days	30.3 [25.0–35.7]
Conventional ventilation, days	7.8 [4.7–10.5]
High-frequency oscillation, days	22.8 [15.7–26.8]
Ibuprofen and/or paracetamol	45 (88.2)
Ibuprofen (first course)	17 (33.3)
Ibuprofen (second course)	28 (54.9)
Paracetamol	14 (27.4)
Diuretics	39 (76.4)
At 36 weeks postconceptional age	
Body weight, g	2328 [2080–2430]
Postnatal growth failure ^a^	11 (22.0)

Data expressed as number (percentage) or median [interquartile range]

^a^ Body weight < -1.28 SD (equivalent to 10th percentile) according to Fenton reference curves. One missing data

Immediately before BTM treatment, 94.1% (*n* = 48) of infants were on invasive ventilation. BTM was administered at a median 28 [26–30] days of life, and the median duration of treatment was 6 [6–9] days. At 24 h after completing BTM treatment, 91.7% (44/48) of infants were weaned from invasive ventilation. Among other infants, who remained on invasive ventilation (8.3%, 4/48; Table 2), one received a second course of BTM. All 3 infants on non-invasive ventilation at the beginning of BTM treatment, reduced their oxygen dependence and capnia (Table 2).

**Table 2:** Postnatal course of premature infants with bronchopulmonary dysplasia and patent ductus arteriosus treated with oral betamethasone

	Total population, *N* = 51
Before betamethasone treatment	
Ventilatory support immediately before treatment	
Invasive ventilation	48 (94.1)
Non-invasive ventilation	3 (5.9)
Weight gain during 7 days before treatment, g/kg/day	17.1 [11.3–22.7]
Betamethasone treatment	
Postnatal age, days	28 [26–30]
Corrected gestational age, weeks	29.8 [26.7–30.8]
Duration of betamethasone treatment, days	6 [6–9]
Total cumulative dose of betamethasone (mg/kg) ^a^	1.38 [1.25–2.4]
Second or subsequent betamethasone treatment	9 (17.6)
During betamethasone treatment	
Systemic hypertension during betamethasone treatment	35 (68.6)
Hyperglycaemia requiring treatment	8 (15.7)
Insulin treatment initiated	5 (9.8)
Increase of pre-existing insulin	3 (5.9)
Glycosuria	6 (11.8)
Weight gain during betamethasone treatment, g/kg/day	5.9 [-4.1–9.5]
After betamethasone treatment	
Respiratory status 24 h after BTM treatment	
In 48 infants on invasive ventilation at the time of BTM	
Weaned from invasive ventilation	44 (91.7)
Still on invasive ventilation	4 (8.3)
In 3 infants on non-invasive ventilation at the time of BTM	
Reduced FiO2 and capnia	3 (100)
Secondary reopening of ductus arteriosus	1 (2.0)
Ligature of PDA	0 (0)
Late-onset sepsis	5 (9.8)
Necrotising enterocolitis grade ≥ 2	5 (9.8)
Weight gain during 7 days after treatment, g/kg/day	21.2 [17.7–27.3]
ΔZ-score for body weight between birth and 36 weeks, SD ^b^	-0.58 [-0.96 – -0.16]

Data expressed as number (percentage) or median [interquartile range]

^a^ One missing data; ^b^ Three missing data

**Fig. 2:**
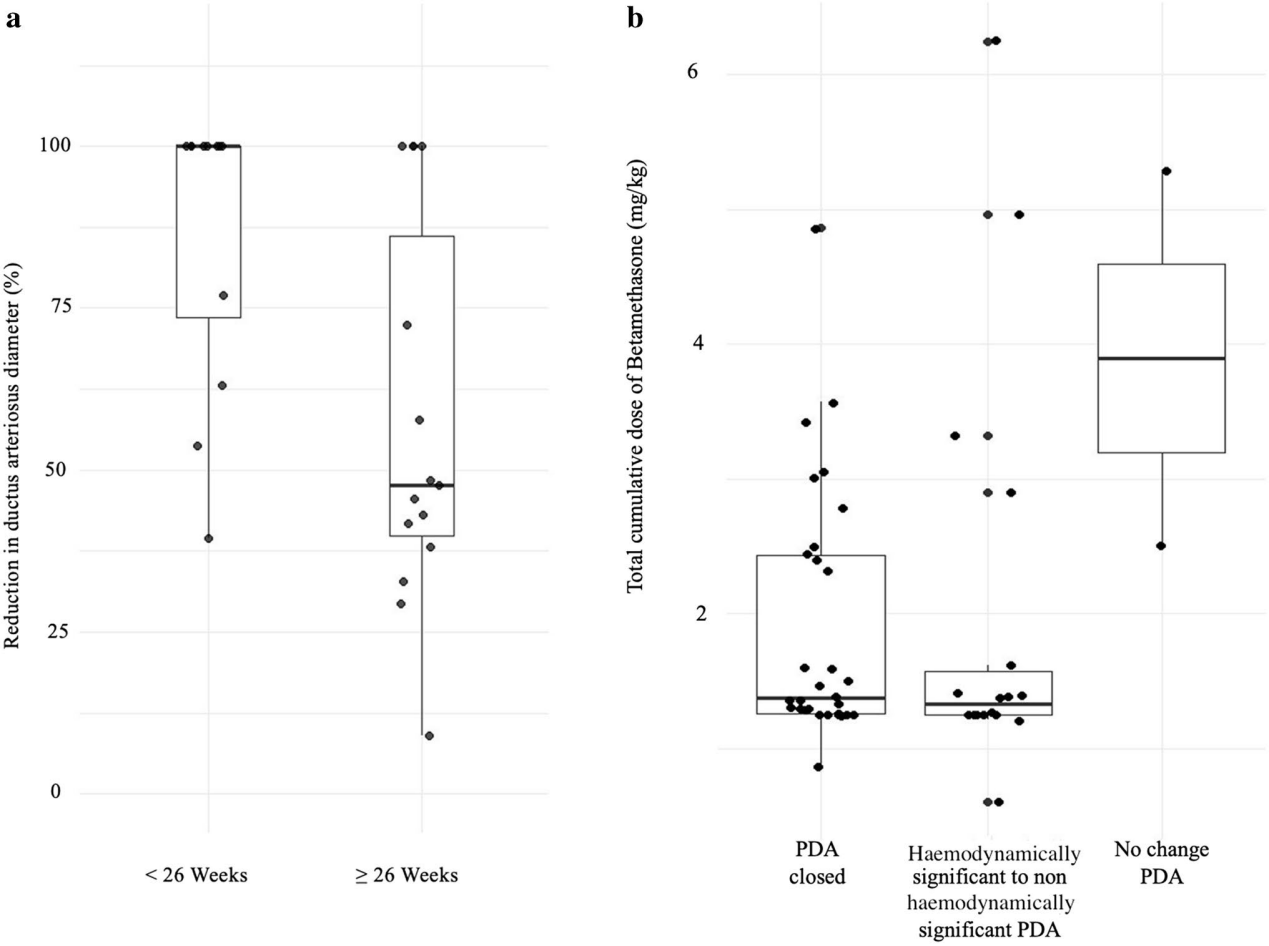
Effect of betamethasone treatment in the 51 extremely premature infants. **a.** Reduction of the diameter of patent ductus arteriosus (PDA) in infants born < 26 or ≥ 26 weeks; significant difference between groups (*p* = 0.015). **b.** Evolution of PDA (closure, evolution from hemodynamically significant to non-hemodynamically significant, or absence of modification). Comparison based on total cumulative dose of betamethasone (in mg/kg). No significant difference between groups (*p* = 0.147). Data are presented as boxplots (median, interquartile range, and extreme values are shown)

At the end of the first course of BTM, 98.0% (*n* = 50) of infants had a closed or a non-haemodynamically significant ductus arteriosus (Table 3). The percent reduction of PDA diameter between the start and the end of BTM treatment was significantly higher in infants with GA < 26 weeks at birth (100% [73.5–100]) than in infants born ≥ 26 weeks (47.7% [39.9–86.2], *p* = 0.015; Fig. 2A). The median total cumulative dose of BTM was not significantly different between infants whose PDA was closed (1.37 [1.26–2.43] mg/kg), those whose PDA became non-hemodynamically significant (1.36 mg/kg [1.25–1.57]), and in the 2 infants who experienced no change in their PDA (3.89 mg/kg [2.50–5.29], *p* = 0.147; Fig. 2B). PDA ligation was not necessary for any infant.

**Table 3:** Patent ductus arteriosus status before and after betamethasone treatment in 51 preterm infants

	Before	After			
		HS	NHS	CLOSED	NHS or CLOSED
HS	41	1	19	21	40
NHS	10	0	1	9	10
Total	51	1	20	30	50

*HS* Haemodynamically significant

*NHS* Non-haemodynamically significant

Two-thirds (68.6%, *n* = 35) of infants had high blood pressure during BTM treatment, but none required anti-hypertensive treatment; 15.7% (*n* = 8) of infants experienced hyperglycaemia, requiring the initiation or increase of insulin treatment during oral BTM treatment. There were 5 (9.8%) infants who experienced LOS after BTM treatment, for 3 of whom this occurred > 2 weeks after BTM treatment; 5 infants experienced NEC (9.8%), for 2 of whom this occurred postoperatively following inguinal hernia repair. The median weight gain during BTM treatment was 5.9 [-4.1–9.5] g/kg/day; during the week before treatment this was 17.1 g/kg/day, during the week before treatment this was 21.2 g/kg/day. At 36 weeks corrected GA, 22% of infants had a body weight below the 10th percentile. The difference in Z-score for weight between birth and 36 weeks of corrected GA was -0.58 [-0.96– -0.16] (Table 2).

## Discussion

In a population of extremely premature infants with PDA and treated with oral BTM to facilitate ventilatory weaning, this, as well as the closure of the PDA, was attained in almost all of the infants just after BTM treatment.

We observed that late BTM treatment had an impact on PDA course which has not been systematically reported in previous studies using late DXM or hydrocortisone. For instance, two retrospective studies in very small number of infants reported PDA closure after the late administration of high-dose intravenous DXM [12, 13]. However, in a randomized controlled study (DART trial) that included 70 infants there was no effect of late, low-dose, DXM on the frequency of PDA (34.3% vs. 42.9% in the placebo group, *p* = 0.46) [9]**.** Concerning hydrocortisone, in a large multicentre randomized trial that included 371 infants there was no significant effect of late, high-dose, hydrocortisone on the frequency of PDA (39.8% vs. 41.1%, *p* = 0.62) [17]. It is of note that this absence of effect of late postnatal steroid treatment is contrary to that reported for early treatment, as the frequency of PDA lower with DXM than placebo (10.6% vs.26.2%, *p* < 0.01 [14]**,** 23% vs. 59%, *p* = 0.05 [15]), or hydrocortisone (36% vs. 73%, *p* = 0.01 [18]) even when this drug was used at a low dose in the PREMILOC study (PDA ligation: 15% vs. 21%, *p* = 0.03 [19]). The impact of late BTM treatment on PDA course observed in the present study could be due to differences in the product used or differences in the cumulative dose. Herein, the cumulative BTM dose was higher (1.38 mg/kg) than the BTM equivalent cumulative dose of DXM in the DART trial (DXM: 0.89 mg/kg, i.e. 0.89 mg/kg BTM equivalent) [9]. However, it was lower than the BTM equivalent hydrocortisone cumulative dose used in the randomized trial that evaluated hydrocortisone (72.5 mg/kg, i.e. 2.7 mg/kg BTM equivalent) [17], suggesting that the effect is unlikely to be related to the dose of steroids used. Concerning the effect of the product used, there is, to our knowledge, no published study has compared DXM, hydrocortisone and BTM to one another with regards to the effect on closing PDA. However, the effectiveness of BTM reported herein could be related to its contractile effect on the ductus arteriosus that has been reported in utero [29], as well as in vitro [22].

Other than the effect on PDA the present study, late oral BTM treatment administered during a short period, at low doses, helped to wean from invasive ventilation, reduce oxygen needs and hypercapnia in extremely preterm infants. Nearly all infants (91.7%) with invasive ventilatory support at the beginning of BTM treatment were weaned from ventilation 24 h after completing BTM treatment. This is a higher frequency of extubation than in randomized trials using DXM (60.0% 10 days after the beginning of DXM) [9] or hydrocortisone (66.3% 14 days after the beginning of hydrocortisone) [17]. Reduction of lung inflammation could improve respiratory function favouring closure of PDA, but data supporting this hypothesis are lacking, and it could be that the closure of the PDA is responsible for the improvement of the respiratory state.

In the present study there were few complications related to the administration of oral BTM. Transient systemic hypertension was present in a little over two-thirds of infants during the BTM treatment, but was moderate, not requiring initiation of anti-hypertensive treatment. Only one in ten infants developed hyperglycaemia treated by insulin treatment during the BTM course, which is close to that we reported in a previous study that included very premature infants treated by BTM (11%) [24]. Another important point is that postnatal DXM treatment is associated with impaired postnatal growth [30]. Herein, the weight gain (5.9 g/kg/day) treatment with BTM is lower than foetal growth (around 15 g/kg/day) [27], which could be explained by protein hypercatabolism induced by glucocorticoids. Nevertheless, the weight gain returned to a normal value quickly after the treatment and the difference in weight Z-score between birth and 36 weeks of corrected GA was low (-0.58 standard deviations, SD), corresponding to the expected value (maximum loss of -0.8 to -1 SD) [31]. Furthermore, the long-term effects of BTM on the neurodevelopment of premature infants was not investigated herein, but in a cohort of extremely premature infants treated with BTM we recently observed the absence of adverse effects on neurodevelopment at 2 years [32], although a randomized trial is needed to confirm this.

The results presented herein suggest that, when PDA is still hemodynamically significant in extremely preterm infants still requiring ventilatory support around 3 weeks of life, it could be beneficial to treat such patients with oral BTM before considering treatment with surgery or endovascular route, which are both associated with severe complications [3].

The present study has, however, certain limitations. Notably, its single-centre nature limited the number of eligible infants. Furthermore, the external validity of the results could be limited as they may not be reproducible with a different corticosteroid therapy protocol or a different management of invasive and non-invasive ventilation than that practiced in our neonatal unit. However, the observation of short-term hemodynamic and respiratory benefits, without major short-term adverse effects, suggest that it could be possible to evaluate oral BTM in the context of prospective studies in larger populations, with long-term follow-up. Another limitation could be the heterogeneity of our population as about 20% presented a non HSPDA. They were included as our main criteria for steroid treatment was developing bronchopulmonary dysplasia, preventing from progressing towards weaning from invasive ventilation or leading to reintubation. We did not exclude them as we hypothesized that, although PDA was non haemodunamiclly significant from an echocardiographic point of view, could represent an additional factor with a particular impact in this specific context.

In conclusion, the use of oral BTM in extremely premature infants with dependence on ventilatory support after 3 weeks of life can help wean invasive ventilation and is associated with PDA closure. Further large multicentre randomized studies could help to optimise the use of postnatal corticosteroid therapy and reduce the use of surgical or endovascular treatment which are associated with serious adverse effects.

## Data Availability

No datasets were generated or analysed during the current study.

## Abbreviations

BPD: Bronchopulmonary Dysplasia
BTM: Betamethasone
D: Day
DXM: Dexamethasone
FiO_2_: Fraction of inspired oxygen
GA: Gestational Age
IVH: Intraventricular Haemorrhage
kPa: Kilo Pascal
LOS: Late-Onset Sepsis
NEC: Necrotising Enterocolitis
NIV: Non-Invasive Ventilation
PCA: Postconceptional Age
PaCO_2_: Arterial carbon dioxide tension
PDA: Patent Ductus Arteriosus
SD: Standard Deviation
